# Subclinical Hypothyroidism in Children: When a Replacement Hormonal Treatment Might Be Advisable

**DOI:** 10.3389/fendo.2019.00109

**Published:** 2019-02-25

**Authors:** Giuseppe Crisafulli, Tommaso Aversa, Giuseppina Zirilli, Giovanni Battista Pajno, Domenico Corica, Filippo De Luca, Malgorzata Wasniewska

**Affiliations:** Department of Human Pathology in Adulthood and Childhood, University of Messina, Messina, Italy

**Keywords:** compensated hypothyroidism, Hashimoto's thyroiditis, idiopathic subclinical hypothyroidism, isolated hyperthyrotropinemia, thyroid status prognosis

## Abstract

Aim of this mini review was to analyze the main variables which should be taken into account when the decision regarding a possible treatment with L-T4 has to be considered for a child with subclinical hypothyroidism (SH). The indications of periodical monitoring and vigilance have been also discussed. It was inferred that therapy should be recommended for children with underlying Hashimoto's thyroiditis and progressive deterioration of thyroid status over time, particularly in the cases with goiter and hypothyroid symptoms and in those with associated Turner syndrome or Down's syndrome and/or other autoimmune diseases. Treatment might also be recommended for children with proatherogenic metabolic abnormalities. Treatment is not advisable in children with idiopathic and mild SH, no goiter, no hypothyroid symptoms and negative anti-thyroid autoantibodies. In the absence of any therapeutic intervention, clinical status and thyroid function tests should be periodically monitored, in order to individuate the children who might benefit from treatment. It has been suggested that children with a persistent mild elevation of TSH, who are not treated with L-T4, should undergo biochemical monitoring of thyroid function and re-assessment of clinical status every 6 months. After 2 years with stable thyroid function tests, the interval between monitoring can be extended.

## Background

Subclinical hypothyroidism (SH) is a biochemical condition where TSH serum levels are above the upper limit of the reference range for the assay, whereas FT4 values are within the reference interval of the assay (1). Depending on the degree of TSH elevation, SH could be defined as either mild or severe, according to whether TSH serum levels range between 4.5 and 10 mIU/l or are >10 mIU/l (2). This condition is also known as isolated hyperthyrotropinemia or compensated hypothyroidism.

The prevalence of SH is especially elevated in older age groups and in women (3), in Caucasians (4) and in populations with high iodine intake (5). Prevalence peak is achieved in women >60 years: 11.6% (6). In children and adolescents SH prevalence seems to be distinctly lower, i.e., <2% (7, 8).

In pediatric age SH etiology may be ascribed to either thyroidal or non-thyroidal causes: Hashimoto's thyroiditis (HT), antiepileptic treatment, celiac disease, cystic fibrosis, chronic renal failure, Turner syndrome (TS), Down's syndrome (DS) and Williams syndrome (9). Obesity is another condition which may be often associated with SH (9). In many cases, however, no definite etiology can be found (idiopathic SH).

The clinical presentation of SH may widely vary, ranging from no manifestations to a clear picture of thyroid impairment (9).

Over time SH may either progress to overt hypothyroidism or regress to euthyroidism. In most cases, however, it remains relatively stable for long periods, at least in the pediatric age and in individuals with idiopathic and mild SH (10–14).

The key-point question in the management of children with SH is whether they should be treated or not, a problem that is still debated, owing to the lack, even in adulthood, of randomized clinical trials revealing significant benefits of L-T4 therapy on life quality, hypothyroid symptoms, heart function and serum lipid levels (15). In pediatric age, this issue is even more controversial (16–18).

The main issue is that children with SH recruited for L-T4 therapy are often maintained on this treatment for many years or lifelong and this is why it is so important to answer the question whether someone has a true thyroid hypofunction and should be treated to the end of life or has only a temporary SH with no long-term relevance. In these cases, treatment should be offered only for some period of time, particularly in obese patients.

Aim of this mini review is to analyze the main variables which should be taken into account when the decision regarding a possible treatment with L-T4 has to be considered for a child with SH. The indications of vigilance and periodical monitoring of thyroid function will be also discussed.

## Baseline TSH Values as Predictors of Thyroid Status Evolution

TSH elevation in the patients with SH is generally interpreted as the biochemical epiphenomenon of a mild thyroid function impairment, with a consequently reduced availability of thyroid hormone at pituitary level (1). On the light of this pathophysiological interpretation of SH, patients with this condition are likely to need increased amounts of TSH to adequately stimulate thyroid gland and a more relevant TSH elevation might, consequently, reflect a more severe impairment of thyroid function (17). On the basis of this view, it can be inferred that baseline TSH values have to be considered the most powerful predictors of the evolution of SH over time, as already postulated by other authors (10, 19, 20).

This inference is supported by the finding that, in a cohort of L-T4 treated children with mild and idiopathic SH, post-therapy TSH outcome is mainly conditioned by baseline TSH levels (17).

Therefore, it is not surprising that L-T4 therapy is currently recommended for TSH levels above 10 mIU/l, whilst such treatment remains a matter of debate for TSH values between 4.5 and 10 mIU/l (1, 13). Furthermore, it has to be emphasized that a large metanalysis of 11 prospective studies on this topic has documented an increased risk of cardiovascular (CV) morbidity and mortality only in SH patients with TSH above 10 mIU/l and no increase in total mortality in any other groups with milder SH, irrespective of TSH values (21).

## Evolution of Thyroid Status Over Time According to the Etiology of SH

The etiology of SH is another factor which can significantly condition the natural course of thyroid function in children with this biochemical condition.

In particular, in children with no underlying pathological disorders, SH has been described as a benign and self-remitting condition (13). In fact, according to the results of the few available follow-up studies on the natural evolution of idiopathic and mild SH in pediatric age, the risk of progression toward overt thyroid failure in these cases seems to be negligible (11–14). Furthermore, the persistence of a SH over time was not found to be associated with either alterations in growth, body mass index, bone maturation and cognitive function or any other problems which could be ascribed to SH, even after 2–5 years with no therapeutic intervention (11, 13, 14).

By contrast, the natural evolution of thyroid status over time has been reported to be significantly more severe in SH children with underlying HT (14, 21–23).

In children HT may present with very different biochemical patterns, ranging from euthyroidism to hyperthyroidism (24, 25). SH is the second most common presentation pattern of HT in childhood, after euthyroidism (24). In the cases with HT-related SH, the risk of a deterioration over time of thyroid function is higher than 50%, whereas the probability of a spontaneous TSH normalization is relatively low, i.e., around 20% (22). On the contrary, in children with mild and idiopathic SH, the risk of progression to overt hypothyroidism is around 11% and the probability of a spontaneous TSH normalization is relatively high, i.e., around 40% (22).

Overall, in two groups of children with either idiopathic or HT-related mild SH (initial TSH 5–10 mIU/l), the percentages of patients who either spontaneously normalize or maintain a stable TSH throughout a 2-year follow-up have been reported to be significantly more elevated in the cohort with idiopathic SH (22). In contrast, the percentage of children whose TSH values increase to >10 mIU/l and require L-T4 treatment was found to be significantly higher in the cohort with HT-related SH (22).

**Figure 1:**
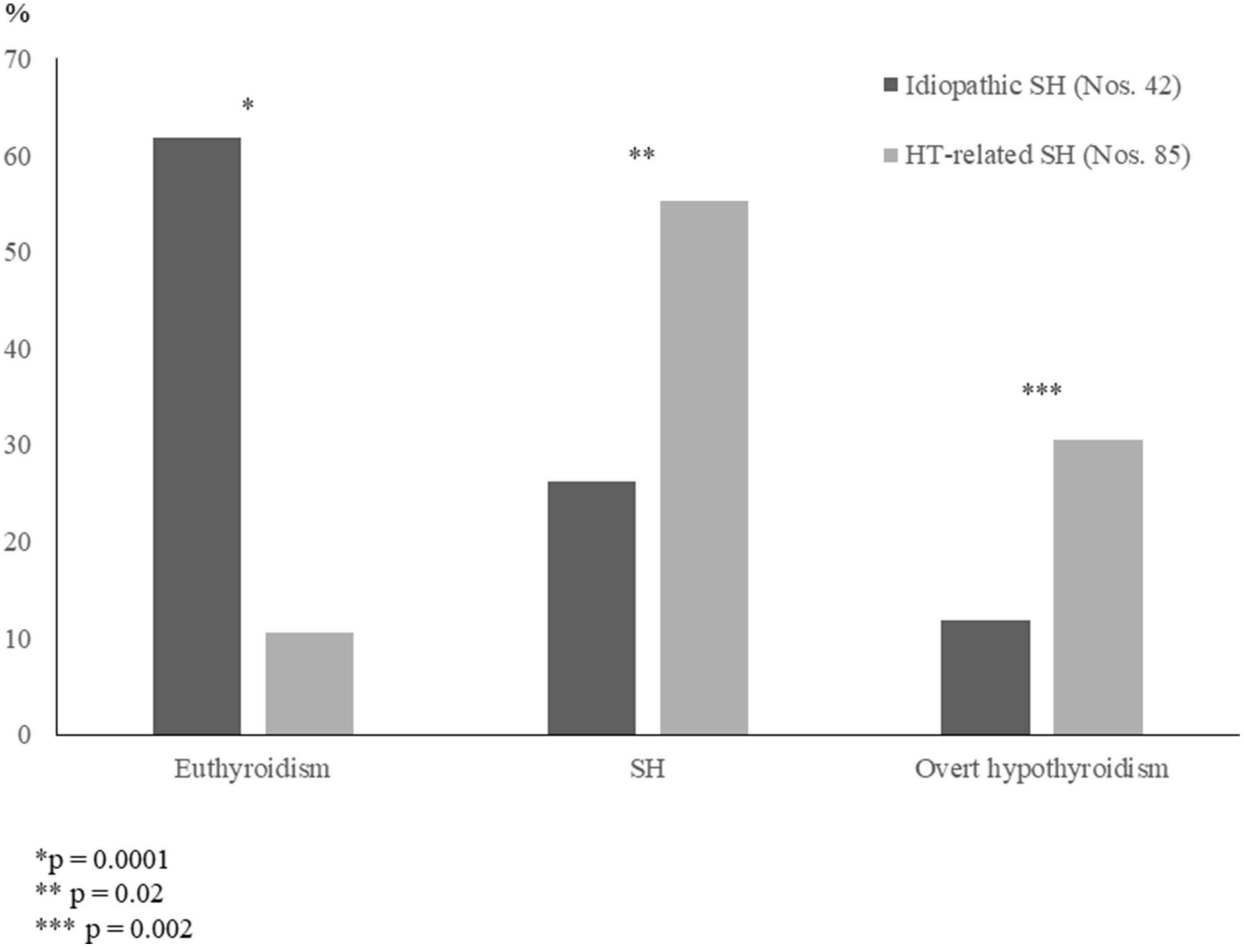
Prevalence (%) of the main biochemical patterns of thyroid function found, at the conclusion of a 5-year follow-up, in two untreated groups of children who had initially presented with either idiopathic subclinical hypothyroidism (SH) or Hashimoto's thyroiditis (HT)-related SH (according to the results of [14] study).

According to the results of another very similar prospective study based on a 5-year follow-up (14), it was confirmed that the long-term prognosis of a mild and idiopathic SH is frequently benign (Figure 1). By contrast, long-term prognosis of thyroid function was found to be significantly more severe in the children with mild but HT-related SH (Figure 1). The association with either TS or DS seems to be able to furtherly impair the outcome of HT-related SH (14).

**Figure 2:**
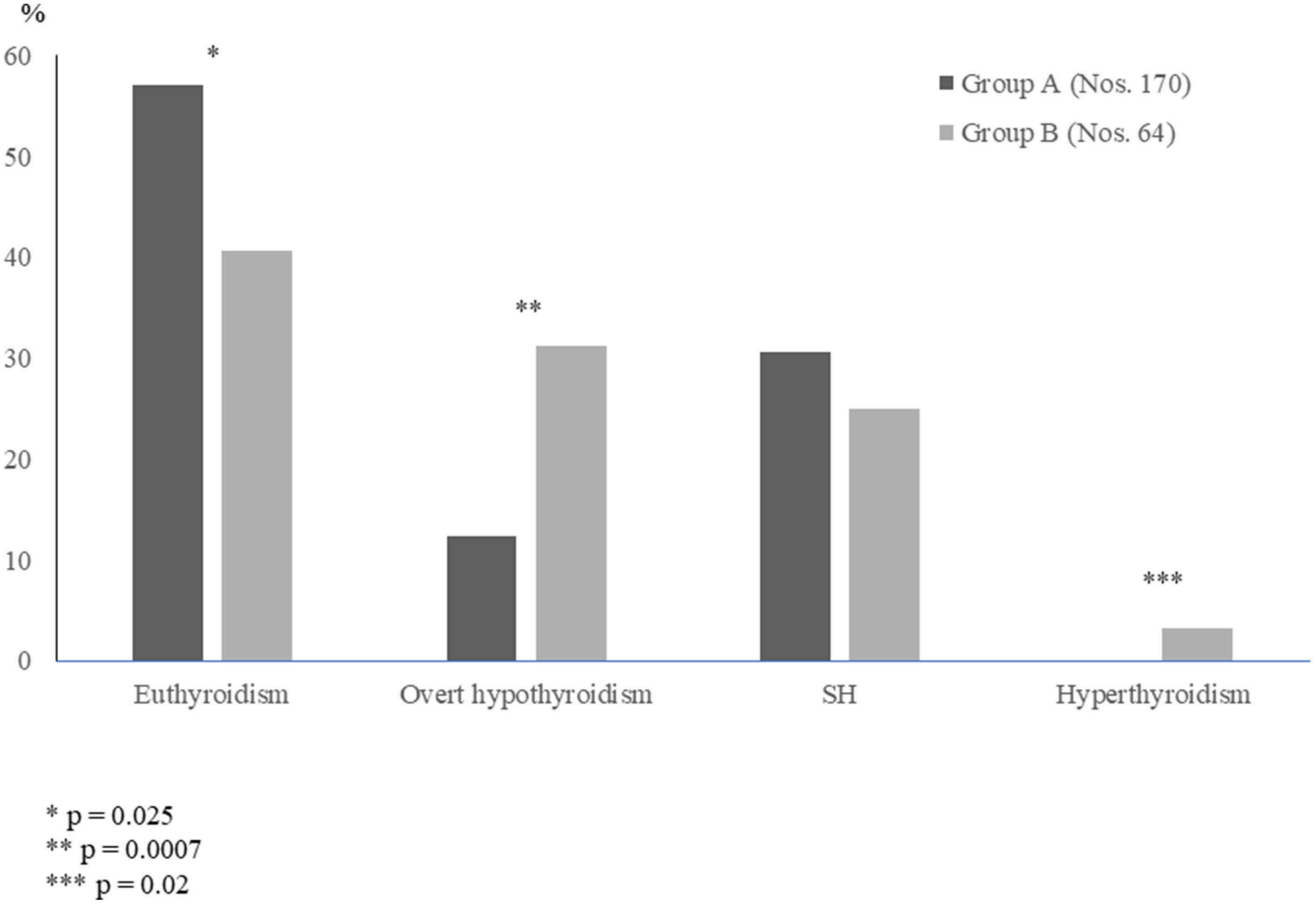
Prevalence (%) of the different biochemical pictures of thyroid function found, at the end of a 5-year follow-up, in two groups of children with Hashimoto's thyroiditis, who had initially presented with either euthyroidism (Group A) or subclinical hypothyroidism SH (Group B) (according to the results of [23] study).

It has been just recently investigated whether the long-term evolution of thyroid function may be different in the HT children who initially presented with either euthyroidism or SH (23), i.e., the two biochemical patterns of thyroid function which are most frequently encountered at diagnosis of juvenile HT (24). According to the results of that study, it may be inferred that the evolution of thyroid status in children with HT is frequently characterized by a spontaneous worsening over time, even in the cases who initially present with a mild biochemical picture (23). In fact, during a 5-year observation period, median TSH significantly increased and mean FT4 significantly decreased in the overall study population (23). When the children who had presented with SH were compared with the ones who were initially euthyroid, the only difference in terms of thyroid function prognosis was that the prevalence of euthyroidism, at the end of follow-up, was significantly higher in the group with initial euthyroidism, whereas the prevalence of either overt hypothyroidism or hyperthyroidism was significantly higher in the other group (Figure 2). These findings confirm the view that children with HT who present with a SH picture might be more prone to develop over time a severe thyroid dysfunction (26). It has to be underlined, however, that the long-term prognosis of thyroid status in children with HT-related SH is not necessarily unfavorable, since 40.6% of the SH patients included in the study by Aversa et al. (23) spontaneously normalized their TSH values at the end of follow-up. Such a percentage of children who became euthyroid over time was not far from that reported by other authors in children with HT-related SH (16, 18, 27).

Among children with HT-related SH, a spontaneous worsening of thyroid status has been reported to occur more frequently in those with celiac disease and/or other associated risk factors than in the ones with no concomitant risk factors (20).

However, treatment with L-T4 in children with HT-related SH should not be under any debate since coexisting thyroid cancer is possible (28–30) and the role of elevated TSH in cancer pathogenesis cannot be missed (31–33).

## Pathological Repercussions of Prolonged SH

Although SH in childhood is often asymptomatic, nevertheless it has to be considered that its clinical expression may widely vary, ranging from no manifestations to clear symptoms of hypothyroidism (18).

Recent studies suggest that untreated children with long-standing SH may develop a cluster of subtle metabolic and proatherogenic abnormalities, such as increased visceral adiposity and slight alterations in lipid profile and homocysteine levels (2, 34). Although the children included in these studies did not develop overt dyslipidemia (2, 34), they exhibited a lipid profile prone to enhancement of atherosclerosis, as documented by slight alterations in HDL-cholesterol (HDL-C) and triglyceride/HDL-C ratio, i.e., two early markers of atherosclerotic disease and cardiometabolic risk even in childhood (35, 36). Moreover, the triglyceride/HDL-C ratio has been recently proposed, in pediatric age, as a helpful index in the prediction of increased arterial stiffness and, therefore, in the selection of children requiring an intervention to prevent atherosclerosis (37). It is important to underline that an atherogenic risk profile in children with SH had been previously reported also by other authors (38, 39).

Owing to the cross-sectional design of the available studies on the relationships between SH and atherogenic abnormalities in pediatric age, it is still controversial whether SH in children is really associated with an increased risk of atherosclerotic disease (40). Nevertheless, in the cases who exhibit during follow-up a deterioration of atherogenic risk profile, a 2-year treatment with L-T4 might be suitable, in order to prevent CV disease in adulthood (34). However, the long-term impact of such therapy on metabolic outcomes in SH children still remains unclear (34).

Other potential clinical repercussions of a prolonged SH in pediatric age regard neurocognitive development, growth, bone maturation, body mass index and thyroid size (41, 42).

According to the study by Cerbone et al. (13), that analyzed growth and intellectual parameters in a series of 36 children with longstanding idiopathic SH, no alterations in growth, bone maturation, body mass index and cognitive functions could be detected, as consequences of persistently elevated TSH serum levels (13). Therefore, it was argued that thyroid hormones involved in growth, bone maturation and neurocognitive development seem to work properly, regardless of persistently increased TSH values, even in the absence of replacement treatment (16). Furthermore, it has to be added that the efficacy of L-T4 therapy on neurocognitive function of children with SH was specifically investigated in the study by Aijaz et al. (43) and no effects on neuropsychological functions were shown during a 2-month period; in particular, no improvement in attention problem was recorded (43).

To the best of our knowledge, the only available report on the effects of L-T4 treatment on thyroid volume in SH children takes into consideration only individuals with HT-related SH (44), whereas no study has hitherto investigated thyroid size changes in treated children with idiopathic SH. According to the results of the study by Svensson et al. (44), L-T4 treatment in children with HT-related SH is able to induce a significant reduction in thyroid size, as also confirmed at ultrasonography evaluation (44).

To sum up, on the light of the available clinical evidence, it may be argued that the only potential beneficial effects of L-T4 therapy in SH children concern a possible improvement of lipid profile in individuals with early markers of atherosclerosis (41) and a reduction of thyroid volume in children with HT-related SH (44).

## When to Treat

According to the consensus statements about the management of SH, L-T4 treatment is recommended in women who are pregnant or who plan pregnancy and in adult patients with TSH persistently above 10 mIU/l (45, 46). In addition, many authors recommend therapy in patients with thyroid enlargement and hypothyroid symptoms or signs (1). However, none of the consensus guidelines published to now addressed the issue of treating or not SH in pediatric populations, which explains why the decision about SH treatment in children and adolescents is still a matter of debate (18).

The optimum management of SH children should take into account the degree of TSH increase and the etiology of thyroid dysfunction and should be individually tailored, considering the severity of the clinical and biochemical abnormalities that are detected in the single cases (41). In fact, whereas the benefits of replacement treatment are clear for the severe forms of SH, the opportunity of such treatment remains uncertain for the mild forms of this disorder (41).

To sum up, treatment should be recommended for children with HT-related SH and progressive deterioration of thyroid status over time, particularly in the cases with associated TS or DS and/or other autoimmune diseases. Treatment might also be recommended for the children with goiter (43) and hypothyroid signs or symptoms and/or proatherogenic metabolic abnormalities (34). Replacement is not indicated in children with idiopathic and mild SH, negative anti-thyroid autoantibodies, no goiter and no evidence of HT at ultrasonography (16). In fact, a 2-year treatment with L-T4 in children with idiopathic and mild SH has been demonstrated to be unable to modify the natural course of SH, at least in terms of post-therapy outcome of hyperthyrotropinemia (17). However, it has to be considered that a small percentage of children with HT may not have thyroid autoantibodies and that, on the contrary, these antibodies may be found even in children without autoimmune thyroid disorders (4, 47).

In the absence of any therapeutic intervention, clinical status and thyroid function tests should be periodically monitored, in order to individuate the children who might benefit from treatment (41). It has been suggested that children with a persistent mild elevation of TSH, who are not treated with L-T4, should undergo biochemical monitoring of thyroid function and re-assessment of clinical status every 6 months (1). After 2 years with stable thyroid function tests, the interval between monitoring can be extended (1).

Finally, one should keep in mind that elevated TSH in children with a thyroid nodule is an independent predictor of thyroid malignancy (31–33) and, therefore, objective parameters such as goiter and ultrasound examination should be crucial in follow-up of SH individuals left untreated, as well as treated but with a history of prior TSH elevation.

## Conclusions

(1) Idiopathic and mild SH in children is generally a benign and self-remitting condition; (2) long-term prognosis may be more severe in the cases with more elevated TSH levels at diagnosis (>10 mIU/l) and in those with underlying HT, especially if associated with TS or DS; (3) SH is often asymptomatic, but goiter and/or subtle proatherogenic metabolic abnormalities may be occasionally detected and might benefit from L-T4 treatment; (4) such therapy is not indicated in asymptomatic children with mild and idiopathic SH, whilst it may be considered in children with HT-related SH and persistent TSH elevation.

## Author Contributions

FD has conceived the paper. TA and MW have organized the material and prepared its distribution in the different sections. GC and MW have written the paper. GZ and DC have prepared the graphics. GP has collected references. Each Author listed on the manuscript has seen and approved the submission of the present version of the manuscript and takes full responsibility for the manuscript.

### Conflict of Interest Statement

The authors declare that the research was conducted in the absence of any commercial or financial relationships that could be construed as a potential conflict of interest. The reviewer GR declared a past co-authorship with several of the authors FD, TA to the handling Editor.

## Abbreviations

C: cholesterol
CV: cardiovascular
DS: Down's syndrome
HT: Hashimoto's thyroiditis
SH: subclinical hypothyroidism
TS: Turner syndrome.
